# Alpha-melanocyte-stimulating hormone contributes to an anti-inflammatory response to lipopolysaccharide

**DOI:** 10.1016/j.molmet.2024.101986

**Published:** 2024-07-09

**Authors:** R.P. Reynolds, R.R. Fan, A. Tinajero, X. Luo, S.C. Huen, T. Fujikawa, S. Lee, A. Lemoff, K.G. Mountjoy, J.K. Elmquist

**Affiliations:** 1 Department of Internal Medicine, Center for Hypothalamic Research, Dallas, TX, USA; 2 Department of Biochemistry, Dallas, TX, USA; 3 Department of Internal Medicine (Nephrology) and Pharmacology, Dallas, TX, USA; 4 The Peter O'Donnell Jr. Brain Institute, University of Texas Southwestern Medical Center, Dallas, TX, USA; 5 Department of Molecular Medicine and Pathology, University of Auckland, Private Bag 92019, Auckland 1043, New Zealand

**Keywords:** LPS, Thermogenesis, POMC, α-MSH, Mouse model

## Abstract

**Objective:**

During infection, metabolism and immunity react dynamically to promote survival through mechanisms that remain unclear. Pro-opiomelanocortin (POMC) cleavage products are produced and released in the brain and in the pituitary gland. One POMC cleavage product, alpha-melanocyte-stimulating hormone (α-MSH), is known to regulate food intake and energy expenditure and has anti-inflammatory effects. However, it is not known whether α-MSH is required to regulate physiological anti-inflammatory responses. We recently developed a novel mouse model with a targeted mutation in *Pomc* (*Pomc*^tm1/tm1^ mice) to block production of all α-MSH forms which are required to regulate metabolism. To test whether endogenous α-MSH is required to regulate immune responses, we compared acute bacterial lipopolysaccharide (LPS)-induced inflammation between *Pomc*^tm1/tm1^ and wild-type *Pomc*^wt/wt^ mice.

**Methods:**

We challenged 10- to 14-week-old male *Pomc*^tm1/tm1^ and *Pomc*^wt/wt^ mice with single i.p. injections of either saline or low-dose LPS (100 μg/kg) and monitored immune and metabolic responses. We used telemetry to measure core body temperature (T_b_), ELISA to measure circulating cytokines, corticosterone and α-MSH, and metabolic chambers to measure body weight, food intake, activity, and respiration. We also developed a mass spectrometry method to measure three forms of α-MSH produced in the mouse hypothalamus and pituitary gland.

**Results:**

LPS induced an exaggerated immune response in *Pomc*^tm1/tm1^ compared to *Pomc*^wt/wt^ mice. Both groups of mice were hypoactive and hypothermic following LPS administration, but *Pomc*^tm1/tm1^ mice were significantly more hypothermic compared to control mice injected with LPS. *Pomc*^tm1/tm1^ mice also had reduced oxygen consumption and impaired metabolic responses to LPS compared to controls. *Pomc*^tm1/tm1^ mice had increased levels of key proinflammatory cytokines at 2 h and 4 h post LPS injection compared to *Pomc*^wt/wt^ mice. Lastly, *Pomc*^wt/wt^ mice injected with LPS compared to saline had increased total α-MSH in circulation 2 h post injection.

**Conclusions:**

Our data indicate endogenous α-MSH contributes to the inflammatory immune responses triggered by low-dose LPS administration and suggest that targeting the melanocortin system could be a potential therapeutic for the treatment of sepsis or inflammatory disease.

## Highlights

• *Pomc*^tm1/tm1^ mice lacking α-MSH were hyper-responsive to low-dose LPS compared with *Pomc*^wt/wt^ mice.
• Following LPS injection, *Pomc*^tm1/tm1^ mice had significantly lower core body temperature compared with *Pomc*^wt/wt^ mice.
• Following LPS injection*, Pomc*^tm1/tm1^ mice had a significant decrease in O_2_ consumption and RER compared with *Pomc*^wt/wt^ mice.
• Following LPS injection, key inflammatory cytokines including leptin were increased in *Pomc*^tm1/tm1^ mice vs. *Pomc*^wt/wt^ mice.

## 1 Introduction

The melanocortin system has been extensively studied and established as a key regulator of whole-body metabolism (see reviews [[1], [2], [3]]). All mammals express melanocortins, a set of cleavage products from the prohormone pro-opiomelanocortin (POMC) that includes alpha-melanocyte-stimulating hormone (α-MSH) and adrenocorticotropin (ACTH) [[1], [2], [3]]. The diversity of POMC-derived products and their roles in a variety of physiological processes make POMC peptides inherently complex to study *in vivo* [1]. However, pharmacological studies identified α-MSH as a key regulator of satiety in the hypothalamus by activating the melanocortin 4 receptor (MC4R) [[4], [5], [6], [7]]. *POMC* or *MC4R* deletion causes a severe metabolic phenotype characterized by severe obesity and hyperphagia in humans and mice [[8], [9], [10], [11]], and *MC4R* haploinsufficiency in humans is the most common monogenic cause of obesity accounting for up to 5% of cases [12,13].

Of note, the role of melanocortins in regulating immune responses has been investigated for longer than their roles in metabolism. The melanocortin ACTH is required for the production of glucocorticoids in rodents and humans through the well-characterized hypothalamic-pituitary-adrenal (HPA) axis that regulates stress and immune responses [14,15]. While glucocorticoids have been extensively studied and used in the clinic to reduce inflammation and suppress the immune system [16,17], less is known about the roles of ACTH itself and its cleavage product α-MSH in regulating inflammation. Work by Lipton and colleagues demonstrated that hypothalamic ACTH and α-MSH infusions had antipyretic effects following a challenge with the prominent proinflammatory cytokine interleukin-1β (IL-1β) that causes fever [18]. Furthermore, circulating α-MSH, ACTH, and corticosteroids increased in rabbits following LPS injection as part of a normal immune response [19], which is similar to the immune response in mice and humans [14,20,21]. Subsequent studies confirmed anti-inflammatory and antipyretic roles for α-MSH in rodents [[22], [23], [24], [25]], but it is not clear if α-MSH is required to mount an anti-inflammatory response. Until recently, a major obstacle in understanding the role of α-MSH in coordinating metabolic and immune responses has been the lack of an adequate animal model. We overcome this obstacle by studying a novel mouse line that specifically deletes α-MSH but preserves all other POMC cleavage products (*Pomc*^tm1/tm1^ mice) [26].

POMC is cleaved into multiple melanocortins, which includes ACTH, α-MSH, and γ-MSH. In humans, β-MSH is also produced from the cleavage of POMC, but β-MSH is not present in rodents [2]. Melanocortins are most highly expressed in the pituitary gland in mice and humans [27], where they are released into circulation and activate one of 5 types of melanocortin receptors (MC1–5R) [2,3,6]. Each melanocortin has a different affinity to each receptor subtype, which allows for melanocortins to have specialized functions. For example, MC2R is only activated by ACTH, and other melanocortin receptors like MC4R have low affinity to γ-MSH but high affinity to α-MSH [2,28]. In mice, most pituitary α-MSH is produced in the pars intermedia (a rudimentary structure in humans) of the anterior lobe [2,27], whereas most pituitary α-MSH in humans is produced in pars intermedia-like cells scattered throughout the pars distalis of the anterior lobe [29,30]. The α-MSH peptides are also produced at much lower levels in the hypothalamus in all mammals, where they function as neurotransmitters and potentially neurohormones to regulate metabolism [3,31]. Most research to date has focused on studying monoacetylated α-MSH (commonly referred to simply as α-MSH), while little is known about the two other forms: desacetyl- and diacetyl-α-MSH. Monoacetylated α-MSH is generally understood to be the most abundant form produced by mouse pituitary melanotrophs while desacetyl-α-MSH is believed to be the most abundant form produced in the brain [32,33]. All three forms of α-MSH are biologically active *in vitro* and *in vivo*, but their potencies and functions can differ [[33], [34], [35]]. We recently reported *Pomc*^tm1/tm1^ mice lacking all forms of α-MSH developed obesity and showed that their obesity was rescued by intracerebroventricular (i.c.v.) infusion of either desacetyl-α-MSH or monoacetyl-α-MSH. This work demonstrated that endogenous α-MSH is required to regulate metabolism and i.c.v. infusion of desacetyl-α-MSH, like α-MSH, regulates body weight [26,36]. Here, we studied *Pomc*^tm1/tm1^ mice to determine whether endogenous α-MSH is required for regulating immune responses, and the potential role of α-MSH in these responses.

We administered lipopolysaccharide (LPS, a gram-negative bacterial cell wall component and a toll-like 4 receptor [TLR4] agonist [37]) to mice lacking α-MSH to study the role of this peptide in regulating immune responses. LPS stimulates immune responses in the host through a well-characterized pathway involving immune cell activation and cytokine production [37,38]. LPS is a commonly used model vs other methods to challenge the immune system in mice due to the ease of administration, controlled dose, and consistency in the responses between mice [39]. We challenged male *Pomc*^wt/wt^ and *Pomc*^tm1/tm1^ mice with intraperitoneal (i.p.) injections of saline (control) or low-dose LPS (100 μg/kg) and studied immune and metabolic responses post-injection. We then adapted a previously published mass spectrometry method [40] to measure total peptide levels of the 3 forms of α-MSH in the mediobasal hypothalamus (MBH, which includes the entire arcuate nucleus) and pituitary gland. Overall, we identified a critical role for melanocortins in coordinating metabolic and immune systems in mice after LPS injection.

## 2 Materials and methods

### 2.1 Animals

Animal work performed in this study was approved and conducted under the oversight of the UT Southwestern Institutional Animal Care and Use Committee (IACUC). *Pomc*^wt/wt^ and *Pomc*^tm1/tm1^ mice were obtained by breeding *Pomc*^wt^^/tm1^ littermates and genotyping offspring as previously described [26]. All mice were housed 2–5 per cage and maintained at UT Southwestern Medical Center at an ambient temperature of 23 ± 1 °C with a 12 h light/dark cycle (lights on 0700–1900). Mice were fed normal mouse chow diet (Harlan, Teklad Global 16% Protein Rodent Diet 2016: 12% kcal from fat, 3 kcal/g). All data were collected from 10- to 14-week-old male mice.

### 2.2 Peptide quantification

Tissues were collected and immediately frozen on dry ice then stored at −80 °C until further processing. The mediobasal hypothalamus (MBH) was removed using a mouse brain matrix and scalpel. The whole brain was placed on its dorsal side into the brain matrix and razor blades were used to remove a section between the end of the optic tracts and the beginning of the brainstem (from about Bregma −1 to −3 mm). The slice was laid coronally, and a scalpel was used to remove the MBH by cutting from the lateral sides of the hypothalamus adjacent to the cerebral cortex to the third ventricle just under the thalamus. This region includes the entire arcuate nucleus (ARH) and some surrounding nuclei such as the ventromedial hypothalamus, dorsomedial hypothalamus, lateral hypothalamus, and periventricular hypothalamus, which may contain α-MSH in axon terminals. The whole pituitary gland was removed from the sella turcica of the sphenoid bone using fine forceps. Frozen tissues were processed using a modified version of a previously reported method [40]. Two mouse MBH were combined into 1 sample for processing due to the low levels of α-MSH known to be expressed in the ARH, and total peptide concentrations calculated by mass spectrometry were divided by 2 to approximate the total peptide levels per mouse. Pituitary glands were processed individually. Guanidine hydrochloride (50 μL of 6M) was added to each frozen sample consisting of 1 pituitary gland or 2 combined MBH in low protein-binding microcentrifuge tubes. The tissues were then pipette-lysed and snap frozen on dry ice. Homogenates were then thawed and frozen a total of 3 times before 200 μL of 80% acetonitrile was added to each sample and mixed by pipette before centrifugation at 12,000 *g* × 5 min at 4 °C. The lower aqueous phase (50 μL) was transferred to a new low protein-binding microcentrifuge tube. Stable heavy-isotopic-labeled MSH peptides were synthesized (by 21st Century Biochemicals, Inc.) with isotopically labeled phenylalanine (^13^C_9_, ^15^C_1_) with purities of >90% for desacetyl- and monoacetyl-α-MSH, and 60% for diacetyl-α-MSH determined by HPLC and used as standards for mass spectrometry. One pmol of each heavy standard (desacetyl-α-MSH: SYSM[+16]EHF[+10]RWGKPV[-1], α-MSH: S[+42]YSM[+16]EHF[+10]RWGKPV[-1], diacetyl-α-MSH: S[+84]YSM[+16]EHF[+10]RWGKPV[-1]) in a total volume of 1 μL of 2% acetonitrile containing 0.1% trifluoroacetic acid was spiked into each 50 uL sample. Next, 4 μL of 0.3% of freshly made hydrogen peroxide in water was added and the samples were incubated for 30 min at room temperature to oxidize methionine residues. Sample volumes were then reduced to less than 10 μL in a SpeedVac. Following this, samples were resuspended in 100 μL of 0.1% formic acid in water and solid-phase extraction was performed using a 96 well Oasis PRIME HLB uElution Plate (Waters). The samples were washed with 200 μL of 0.1% formic acid, followed by 200 μL of 5% methanol containing 1% acetic acid, before the peptides were eluted with 2 × 30 μL of 60% methanol containing 10% acetic acid. Peptide eluates were then completely dried in a SpeedVac and reconstituted in 10 μL of 2% acetonitrile containing 0.1% trifluoroacetic acid for selected reaction monitoring (SRM) mass spectrometry analysis.

SRM analysis was performed on an AB Sciex 6500 QTRAP mass spectrometer in positive-ion low-mass mode, using a OptiFlow source with an OptiFlow Nano Electrode (100 nl/min – 1 μL/min, P/N: 5070382). The source settings were as follows: curtain gas = 20, ion spray voltage = 3400, ion source gas 1 = 20. Analyst Software v.1.6 was used to run the mass spectrometer. Reconstituted samples (100 μL) were separated on a Dionex Acclaim PepMap100 reverse-phase C18 column (75 μm × 15 cm) using an Ultimate 3000 RSLCnano HPLC system. The HPLC was controlled using Chromeleon Xpress (version 6.8 SR10) and Dionex Chromatography MS Link v. 2.12. Separation of peptides was carried out at 250 nL/min using a gradient from 5% to 35% B for 15 min, 35%–50% B for 4 min, and 50%–80% B for 5 min, where mobile phase A was 2% acetonitrile (ACN), 0.1% formic acid in water and mobile phase B was 80% ACN, 10% trifluoroethanol, 10% water, 0.1% formic acid. The SRM method monitored the top seven transitions for each form of the MSH peptide and its corresponding heavy-isotope-labeled standard. These transitions were determined by monitoring peak areas for all singly and doubly charged b and y ions below *m*/*z* = 1,250 and all doubly and triply charged peptide ions, and for a mix of the heavy-labeled peptide standards. These data were analyzed using Skyline v4.2 software (http://skyline.maccosslab.org). Collision energies were optimized by subsequent sample injections. Transitions that had interference from impurities or noise peaks were not included when performing peptide quantification.

### 2.3 LPS challenge

Mice were group housed for all experiments except metabolic cage and telemetry experiments, which required single housing. All experiments were performed either at room temperature or 23 °C in temperature-controlled metabolic cages and the LPS used was made up from the same stock of *Escherichia coli* O55:B5 (Sigma Aldrich L2880). The LPS stock solution was reconstituted in saline to a concentration of 1 mg/mL, aliquoted, and stored at −80 °C. LPS aliquots were thawed, diluted further in saline to a working solution, and injected i.p. with a dose of 100 μg/kg for all experiments. Control mice were injected i.p. with saline. Injection volumes for all experiments and treatments were 1% of the animal's body weight (e.g., 0.25 mL for a 25 g mouse).

### 2.4 Hormone and cytokine measurements

Mice were euthanized by decapitation in a dedicated room to minimize stress to the animals between 2 PM and 5 PM for all experiments. Trunk blood was collected into EDTA coated tubes and centrifuged (2000 *g* × 15 min at 4 °C) to extract plasma and stored at −80 °C until analysis. Circulating cytokines (Bio-Rad, M60009RDPD), leptin (Crystal Chem, 90030), and total α-MSH peptides (Mybiosource, MBS2563826) were measured using commercial ELISA kits following manufacturer's instructions. Plasma was shipped on dry ice to Vanderbilt University Hormone Assay & Analytical Services Core for corticosterone quantification.

### 2.5 Telemetry

*Pomc*^wt/wt^ and *Pomc*^tm1/tm1^ mice were anesthetized with 3% isoflurane in a chamber before being transferred to a nose cone and maintained on 2% isoflurane for surgeries. Mice were injected subcutaneously with Buprenorphine SR (0.5 mg/mL, 50 μL per 25 g mouse) and Carprofen (1.3 mg/mL, 100 μL per 25 g mouse) into the interscapular region and rested on a heating pad with eye lubricant applied. The abdominal surgical region was shaved, and excess hair was removed with Nair hair removal cream for 30 s. The surgical field was sterilized using 3 alternating applications of Betadine and ethanol wipes. A horizontal incision was made through the skin and muscle layers and the telemeter (model TA-F10, Data Sciences International, Minnesota, USA) was inserted i.p. and slid laterally towards the bladder and away from vital organs. The muscle layer was then sutured with absorbable 4-0 sutures and the skin was closed with wound clips. After surgery, each mouse was placed on a warming pad in a new cage and given 30 min to recover before the cage was moved back to the housing room. Mice were given an additional injection of Carprofen analgesic every 24 h and checked twice per day, up to 72 h post-surgery. All mice were singly housed after surgery and throughout the telemetry experiment.

After the surgery, mice were given 7–10 days to recover before being moved to a dedicated telemetry room for recordings. Mice were habituated in the telemetry room for at least 24 h before commencing baseline recordings. Telemetry plate receivers were placed under each cage, and up to 12 mice were recorded simultaneously. Injections on experimental Days 1 and 2 (after the 24 h habituation period) were performed 1 h before lights off. Telemetry data were recorded for another 24–48 h after Day 2 injections before mice were euthanized. Telemeters recorded 10 s of core body temperature (T_b_) and locomotor activity every 10 min during the entire recording period.

### 2.6 Metabolic cages

A combined indirect calorimetry system (CaloSys Calorimetry System, TSE Systems Inc.) was used for all metabolic studies. Individually housed mice were acclimated for 5 days in a metabolic chamber with food and water before their cages were moved to recording positions. Oxygen consumption (VO_2_), carbon dioxide production (VCO_2_), food intake, and water intake were then measured for 10 days. Respiratory exchange ratio (RER) was calculated from VO_2_ and VCO_2_ values [41]. On Day 3 of the 10-day testing period, mice were injected i.p. with saline. On Day 6, mice were injected i.p. with 100 μg/kg LPS. All i.p. injections were performed 1 h before lights off.

### 2.7 Statistical analysis

A detailed list of the statistical analyses performed is found in Table S1. Data are expressed as mean ± SEM. Comparisons between two experimental conditions were analyzed by Student's unpaired t-test. Comparisons between more than two experimental conditions were analyzed by Two-way ANOVA followed by Šidák post hoc test. Outlying data were identified using the ROUT method (Q = 1%) and removed from analysis. All statistical tests were performed using GraphPad Prism (Version 10) and *P* < 0.05 was considered statistically significant.

## 3 Results

### 3.1 Validation of α-MSH depletion using mass spectrometry

In order to determine the efficacy of the depletion of all three forms α-MSH in *Pomc*^tm1/tm1^ mice, we modified a method [40] to quantify total peptide levels using SRM mass spectrometry analysis in the MBH and pituitary gland. Surprisingly, we found that in control mice, diacetyl-α-MSH was the most highly produced form in both the MBH and pituitary gland (Figs. S1A–B). As expected, we also found a robust depletion of all three forms of α-MSH in both tissues in *Pomc*^tm1/tm1^ mice (Fig. S1), further validating the functional knockout of all α-MSH forms by the targeted mutation in the *Pomc* gene in *Pomc*^tm1/tm1^ mice.

### 3.2 *Pomc*^tm1/tm1^ mice are hyper-responsive to LPS injection

**Figure 1:**
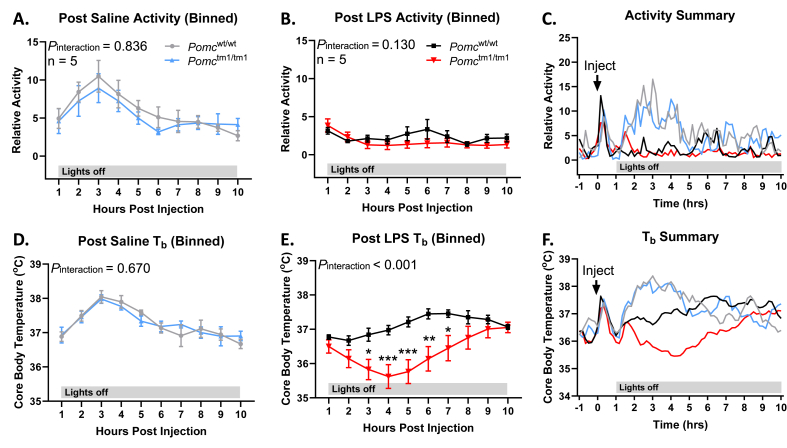
**LPS reduced activity and core body temperature in *Pomc***^**wt/wt**^**and *Pomc***^**tm1/tm1**^**mice.***Pomc*^wt/wt^ and *Pomc*^tm1/tm1^ mice were injected (i.p.) with saline on Day 1 **(A)** and 100 μg/kg LPS on Day 2 **(B)** 1 h before lights off, and relative activity and core body temperature was recorded by telemeter every 10 min. Activity data is binned in 1 h blocks. **(C)** Summary figure of all 10 min recordings for Days 1 and 2 superimposed. Core body temperature was also recorded after i.p. injections of saline on Day 1 **(D)** and 100 μg/kg LPS on Day 2 **(E)**. Summary data are shown for Days 1 and 2 superimposed **(F)**. The data are expressed as the mean ± SEM. ∗p < 0.05 compared to littermate controls.

Baseline activity was similar in *Pomc*^wt/wt^ and *Pomc*^tm1/tm1^ mice following saline injection on Day 1 (Figure 1A), marked by a normal increase in locomotion during their active phase (lights off) [42]. However, activity dropped markedly (∼50%) in both *Pomc*^wt/wt^ and *Pomc*^tm1/tm1^ mice injected i.p. with 100 μg/kg LPS on Day 2 during the acute phase of the immune response (0–10 h post-injection), as part of the normal sickness response induced by LPS (Figure 1B–C) [43]. T_b_ was also similar in *Pomc*^wt/wt^ and *Pomc*^tm1/tm1^ mice on Day 1 after saline injection and increased normally during their active phase (Figure 1D) [42]. On Day 2, LPS injection induced an acute hypothermic response in both *Pomc*^wt/wt^ and *Pomc*^tm1/tm1^ mice (Figure 1E–F). Strikingly, T_b_ in *Pomc*^tm1/tm1^ mice dropped nearly 2° lower than *Pomc*^wt/wt^ control mice following low-dose LPS injection, suggesting that *Pomc*^tm1/tm1^ mice are hyper-responsive to 100 μg/kg LPS. Differences in activity and T_b_ were not observed following the acute phase for up to 30 h post LPS (Fig. S2). Together, these data show that *Pomc*^tm1/tm1^ mice exhibit an exaggerated acute LPS-induced thermoregulatory response compared to *Pomc*^wt/wt^ mice.

### 3.3 *Pomc*^tm1/tm1^ mice have impaired metabolism following LPS injection

**Figure 2:**
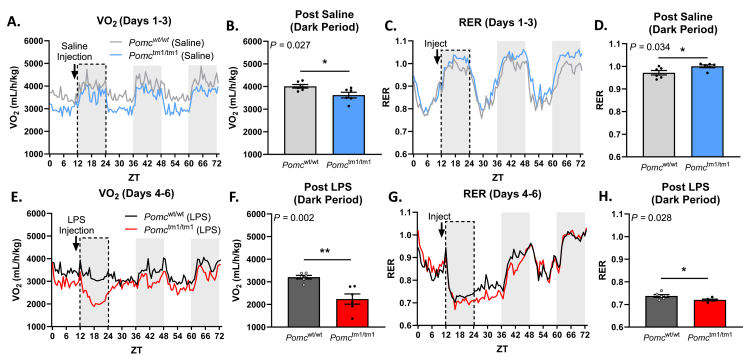
***Pomc***^**tm1/tm1**^**mice had reduced oxygen consumption and RER after LPS injection. (A**–**D)***Pomc*^wt/wt^ and *Pomc*^tm1/tm1^ mice were injected (i.p.) with saline 1 h before lights off on Day 1. VO_2_ was measured and RER calculated for 3 days. **(E–H)***Pomc*^wt/wt^ and *Pomc*^tm1/tm1^ mice were injected i.p. with 100 μg/kg LPS. Gray boxes indicate dark cycle, and bar graphs show average values per mouse for the entire dark period after saline or LPS injection (dotted line region on the line graphs). The data are expressed as the mean ± SEM. ∗p < 0.05, ∗∗p < 0.01 compared to littermate controls.

We next challenged mice with LPS in metabolic cages to measure other aspects of sickness behavior (Figure 2). Oxygen consumption and respiratory exchange ratio (RER) are used as indicators of macronutrient preference; higher RER indicates a preference towards carbohydrate energy utilization while a lower RER indicates a preference towards lipids as an energy substrate [41]. These measurements are important in the context of immune responses as many immune cells are sensitive to and modified by macronutrients [[44], [45], [46]]. Furthermore, reduced oxygen consumption is an independent predictor of mortality in critically ill patients and is therefore of clinical significance [47].

After saline injection, *Pomc*^tm1/tm1^ mice decreased oxygen consumption (Figure 2A–B) which resulted in an elevated respiratory exchange ratio (RER) compared to *Pomc*^wt/wt^ mice, indicating the preferential use of glucose over lipids as an energy substrate in *Pomc*^tm1/tm1^ mice [41](Figure 2C–D). However, *Pomc*^tm1/tm1^ had drastically reduced oxygen consumption following LPS injection (Figure 2E–F), marked by a 38% decrease from post–saline dark period VO_2_ compared to a 20% decrease in *Pomc*^wt/wt^ mice. Furthermore, while RER was elevated in *Pomc*^tm1/tm1^ mice after saline treatment, LPS administration induced a change in energy preference, indicating *Pomc*^tm1/tm1^ mice may rely more on lipids as an energy substrate compared to *Pomc*^wt/wt^ littermate controls during immune responses (Figure 2G–H). Increasing fatty acid metabolism is a typical response in mice and humans during infection [[48], [49], [50], [51]], and may indicate an exaggerated immune response in *Pomc*^tm1/tm1^ mice. This metabolic shift lasted up to 36 h after LPS treatment, and this was not due to a difference in food intake in LPS-treated groups (Fig. S3). Taken together, these data suggest α-MSH is an important regulator of baseline metabolism and metabolic adaptations in response to LPS that may influence the ability of the immune system to properly initiate an immune response.

### 3.4 Peripheral α-MSH increased in *Pomc*^wt/wt^ mice while *Pomc*^tm1/tm1^ mice had normal corticosteroid production but increased leptin following LPS injection

**Figure 3:**
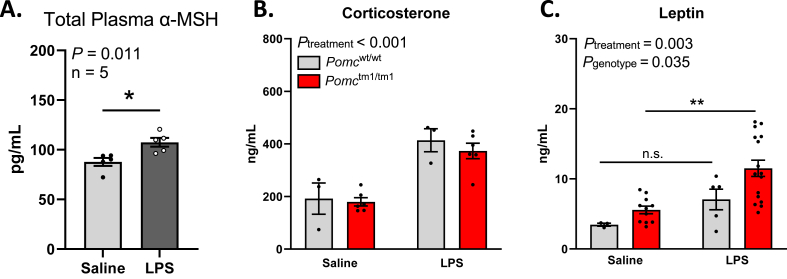
**Peripheral α-MSH increased in *Pomc***^**wt/wt**^**mice and *Pomc***^**tm1/tm1**^**mice had normal corticosteroid production but increased leptin after LPS injection.***Pomc*^wt/wt^ and *Pomc*^tm1/tm1^ mice were injected with 100 μg/kg LPS and plasma was collected 2 h later. **(A)** Total α-MSH (all 3 forms combined) was measured by ELISA in *Pomc*^wt/wt^ mice. **(B)** Corticosterone and **(C)** leptin were measured in *Pomc*^wt/wt^ and *Pomc*^tm1/tm1^ mice by ELISA. The data are expressed as the mean ± SEM. ∗p < 0.05, ∗∗p < 0.01 compared to littermate controls.

Adrenal-derived glucocorticoids (corticosterone in mice, cortisol in humans) are a central part of the immune and stress responses following an infection [52,53]. Others have shown that LPS administration in rodents causes an increase in pituitary gland *Pomc* expression and corresponding increases in circulating ACTH, α-MSH, and glucocorticoid production [14,19,54]. There is currently no method to measure individual forms of α-MSH in circulation, but commercial ELISA kits likely detect total (three forms combined) α-MSH. We found that LPS induces an increase in total circulating α-MSH in *Pomc*^wt/wt^ mice 2 h post-injection (Figure 3A), suggesting this protein may be produced in response to an infection and potentially involved in immune responses.

We next sought to determine whether *Pomc*^*tm1/tm1*^ mice may have impairments in other aspects of their immune responses related to the mutation in *Pomc* in our mouse model. ACTH is a key POMC-derived hormone involved in the synthesis of corticosterone, a key hormone in regulating the immune system, and peaks 2 h after LPS injection in rodents and functions to prevent excess inflammation [55,56]. We have shown previously that male *Pomc*^tm1/tm1^ mice have normal baseline corticosterone levels similar to *Pomc*^wt/wt^ controls, and that the mutated ACTH in these mice still binds and activates MC2R *in vitro* [26]. However, antibody-based techniques that target ACTH are ineffective with our mouse model due to the location of the targeted mutation in *Pomc*^tm1/tm1^ mice. In our model, we mutated the cleavage site on the ACTH protein which is also the epitope-binding site targeted by antibody-based techniques; we therefore cannot measure ACTH in *Pomc*^tm1/tm1^ mice. Instead, we measured the downstream hormone, corticosterone, to assess a component of the early HPA axis response. We found that *Pomc*^*tm1/tm1*^ and *Pomc*^*wt/wt*^ mice have similar concentrations of corticosterone 2 h post-LPS (Figure 3B). These data suggest that both *Pomc*^wt/wt^ and *Pomc*^tm1/tm1^ mice have intact acute glucocorticoid responses to low-dose LPS.

We have previously shown that *Pomc*^tm1/tm1^ mice have elevated leptin due to increased fat mass [36]. Leptin is a critical metabolic hormone produced and released by white adipose tissue and exerts its effects by binding to receptors in the hypothalamus [57,58]. As fat mass increases, circulating levels of leptin also increase, and this hyperleptinemia is predicted to result in the chronic low-grade inflammation and susceptibility to inflammatory disease observed in obese patients [59]. However, this low-grade inflammation is also due to leptin's role as a proinflammatory cytokine which is induced after LPS administration [60,61]. Leptin regulates the immune system [62] and is expressed in immune cells [[63], [64], [65]]. Activation of the leptin receptor increases phagocytic action of macrophages [66] and promotes proinflammatory cytokine secretion [67,68](for review, see [69]). Given the increased body mass (Fig. S4), which we have previously shown is due to increased fat mass and corresponding leptin concentrations in *Pomc*^tm1/tm1^ mice [36], we measured leptin 2 h post LPS injection and found that leptin levels were significantly increased compared to *Pomc*^*wt/wt*^ mice (Figure 3C). These data suggest mice lacking α-MSH may have impaired leptin regulation following an LPS injection, which could contribute to the exaggerated immune responses seen here.

### 3.5 Key proinflammatory cytokines are increased in *Pomc*^tm1/tm1^ mice following LPS injection

**Figure 4:**
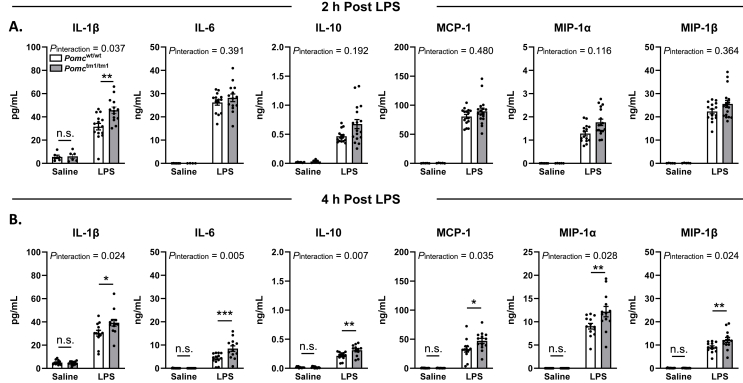
***Pomc***^**tm1/tm1**^**mice had increased cytokine levels compared to *Pomc***^**wt/wt**^**mice 2 h and 4 h after LPS injection.** Plasma from *Pomc*^wt/wt^ and *Pomc*^tm1/tm1^ mice were collected 2 h **(A)** and 4 h **(B)** after 100 μg/kg i.p. LPS injection and cytokine levels were quantified using a cytokine multiplex assay. The data are expressed as the mean ± SEM. ∗p < 0.05 and ∗∗p < 0.01 compared to littermate controls.

Cytokines signal the brain to shift metabolism and behavior to promote survival after infection [70,71]. In gram-negative infections, cytokine production is stimulated via activation of TLR4, primarily through monocytes and macrophages [[72], [73], [74]]. POMC cells in the hypothalamus and pituitary express numerous cytokine receptors and increase *Pomc* mRNA expression after LPS or IL-1β injection [22,[75], [76], [77]]. However, cytokine production must be tightly regulated since excessive cytokine production can be life-threatening, as seen in septic shock [78,79]. Therefore, we determined whether endogenous α-MSH regulates cytokine expression at 0.5 h, 2 h, and 4 h after saline or low-dose LPS injection (100 μg/kg). LPS injection robustly increased cytokine expression in both *Pomc*^wt/wt^ and *Pomc*^tm1/tm1^ mice (Figure 4 and Table S2), consistent with an LPS immune challenge [80]. However, we found that *Pomc*^tm1/tm1^ mice had significantly greater expression of multiple proinflammatory cytokines and chemokines at 2 h and 4 h post-LPS injection (Figure 4). *Pomc*^tm1/tm1^ mice had significantly higher concentrations of IL-1β at 2 h and 4 h post-LPS, and significant increases in IL-6, IL-10, monocyte chemoattractant peptide 1 (MCP-1), and macrophage inflammatory peptides 1α and 1β (MIP-1α and MIP-1β, respectively) at 4 h compared to LPS-injected *Pomc*^wt/wt^ controls (Figure 4). There were no significant differences between *Pomc*^tm1/tm1^ and *Pomc*^wt/wt^ controls 30 min after either saline or LPS injection (Table S2). Together, these data indicate α-MSH is an important regulator of cytokine production in response to LPS, and that mice lacking α-MSH are susceptible to excessive inflammation.

## 4 Discussion

Collectively, our results support the hypothesis that α-MSH regulates inflammatory immune responses triggered by LPS administration [26]. Compared to *Pomc*^wt/wt^ littermates, *Pomc*^tm1/tm1^ mice exhibited an exaggerated immune response to low-dose i.p. LPS, characterized by acute hypothermia, an impaired metabolic response, and increased proinflammatory and inflammatory-related cytokines including leptin. Using ELISA, we show that LPS treatment significantly increased total plasma α-MSH in *Pomc*^wt/wt^ mice 2 h post LPS. These data suggest that α-MSH release, in concert with other immune responses, reduces systemic inflammation to LPS by regulating cytokine production. Without α-MSH, some proinflammatory cytokines are produced in excess following treatment with LPS, which are associated with the exaggerated sickness behaviors observed here in *Pomc*^tm1/tm1^ mice. Furthermore, the lack of hypothalamic α-MSH may contribute to the impaired metabolic responses in *Pomc*^tm1/tm1^ mice characterized by reduced O_2_ consumption and lower RER, and attenuated thermogenesis (resulting in hypothermia and a failure to regulate T_b_) compared to *Pomc*^wt/wt^ mice after LPS injection.

Macrophages and other immune cells express TLR4, LEPR, and melanocortin receptors in mice and humans [63,64,73,81,82]. *In vitro* studies have shown that activation of melanocortin receptors on macrophages inhibits cytokine release via suppression of NF-kB and nitric oxide [[83], [84], [85]], and MC1R [[86], [87], [88], [89]] and MC3R [90] agonists have been shown to reduce inflammation through this same pathway. Indeed, murine macrophages express MC1R [84,89,91], MC3R [92], and MC5R [93], which all bind α-MSH and reduce inflammation. Furthermore, central melanocortin signaling plays a critical role in regulating fever [19,94] as well as central [[95], [96], [97]] and peripheral [81,98,99] immune responses. These responses involve microglia, neurons, and astrocytes, all of which express the melanocortin receptors [97,[100], [101], [102]]. We found that macrophage-derived cytokines, IL-1β, IL-6, IL-10, MCP-1, MIP-1α, and MIP-1β were significantly increased in *Pomc*^tm1/tm1^ mice following low-dose LPS injection compared to LPS-injected *Pomc*^wt/wt^ control mice. Leptin, another important cytokine and adipokine known to regulate inflammation [60,61], was also significantly increased after LPS injection in our study. The primary targets for leptin are the LEPR-expressing hypothalamic neurons (including POMC-expressing ARH neurons) which also express cytokine receptors and are activated after LPS treatment [103,104]. Thus, the hypothalamus is poised to orchestrate metabolic and behavioral responses to immune challenges through highly interconnected networks linked to every metabolic function associated with the sickness behaviors observed in this study, including activity, food intake, energy expenditure, and thermoregulation [105]. α-MSH release in the periphery, along with feedback from peripheral inflammatory signals such as cytokines and leptin to hypothalamic neurons, may be critical in regulating thermogenesis and sickness behaviors following LPS administration.

While these findings agree with and build upon previous literature, there are important limitations to consider. First, while we have modified a method enabling measurement of three forms of α-MSH in eluate from tissue lysate, further work is required to enable quantitative measurement for these forms of α-MSH in animal tissue and to determine their respective roles in response to inflammation. Future matrix-assisted laser desorption/ionization (MALDI)-imaging mass spectrometry to map cell expression of these α-MSH forms in the pituitary gland and hypothalamus will also help to resolve the regulation of α-MSH forms following LPS treatment. Second, our study was limited to acute immune responses specifically to LPS, mediated via the TLR4-dependent pathway, which is commonly used as a model of Gram-negative sepsis [37]. Our studies used low-dose LPS due to the risk of severe sickness and mortality from higher doses of LPS in a mouse model that was hypothesized to have an impaired immune response [46,106]. Our model relied on modeling bacterial inflammation using LPS, but α-MSH may have other effects (or not be involved) in bacterial clearance or other types of responses, such as adaptive or T-cell-mediated immune responses [44,45]. Future studies using alternative methods such as cecal ligation and puncture to model live bacterial infections, as well as injection of different inflammatory pathogens (e.g., viral proteins) and/or ambient temperatures will further elucidate the role of α-MSH in host defense. Lastly, it is unclear from these data if the dysregulated metabolism in *Pomc*^tm1/tm1^ mice contribute to, or cause, the exaggerated immune responses observed here: more studies will be needed to untangle the interactions between metabolic and immune responses in *Pomc*^tm1/tm1^ mice.

Our data support the model that melanocortin agonists could be useful in the context of sepsis and obesity, where leptin and melanocortin signaling may already be deficient. In fact, the side effects of hypertension encountered for treatment of obesity with MC4R agonist [107,108] could also be beneficial in treating refractory septic shock. Furthermore, α-MSH agonists may avoid the undesired side effects of alternative immunosuppressive therapies for sepsis, such as glucocorticoids [109]. Lastly, preclinical studies have highlighted the striking effects of metabolic state on mortality to bacterial and viral infections [46]. Together, these data highlight the interaction between metabolism and inflammation and incentivize further studies that investigate targeting the melanocortin system to treat inflammation.

## 5 Conclusion

In conclusion, we demonstrate a role of the POMC derivative, α-MSH, in mediating immune responses to LPS. Deletion of three forms of α-MSH in male C57BL6/J mice results in exaggerated immune responses to low-dose LPS, characterized by hypothermia, altered metabolism, and increased circulating proinflammatory and inflammatory-related cytokines including leptin. We developed a mass spectrometry method to distinguish between three forms of endogenous α-MSH to better understand α-MSH regulation in health and disease. Our model predicts that α-MSH is released in response to an immune challenge and prevents excess cytokine production to properly tune the physiological response to pathogens. We believe these data warrant further translational studies exploring the therapeutic potential of α-MSH mimetics that target both metabolic and immune responses.

## CRediT authorship contribution statement

**R.P. Reynolds:** Writing – review & editing, Writing – original draft, Visualization, Methodology, Investigation, Formal analysis, Data curation, Conceptualization. **R.R. Fan:** Writing – review & editing, Methodology, Investigation, Formal analysis, Data curation. **A. Tinajero:** Methodology. **X. Luo:** Writing – original draft, Methodology, Formal analysis. **S.C. Huen:** Writing – review & editing, Methodology. **T. Fujikawa:** Writing – review & editing, Writing – original draft. **S. Lee:** Supervision, Conceptualization. **A. Lemoff:** Writing – review & editing, Writing – original draft, Validation, Supervision, Methodology, Formal analysis. **K.G. Mountjoy:** Writing – review & editing, Writing – original draft, Supervision, Methodology, Funding acquisition, Conceptualization. **J.K. Elmquist:** Writing – review & editing, Writing – original draft, Supervision, Resources, Funding acquisition, Conceptualization.

## Declaration of competing interest

The authors declare that they have no known competing financial interests or personal relationships that could have appeared to influence the work reported in this paper.

## Data Availability

Data will be made available on request.
