# Supplementary figures and images for: Alpha-melanocyte-stimulating hormone contributes to an anti-inflammatory response to lipopolysaccharide
Source: Mol Metab. 2024 Jul 9;87:101986. doi: 10.1016/j.molmet.2024.101986 (PMC11362619; doi:10.1016/j.molmet.2024.101986)

## Slide 1
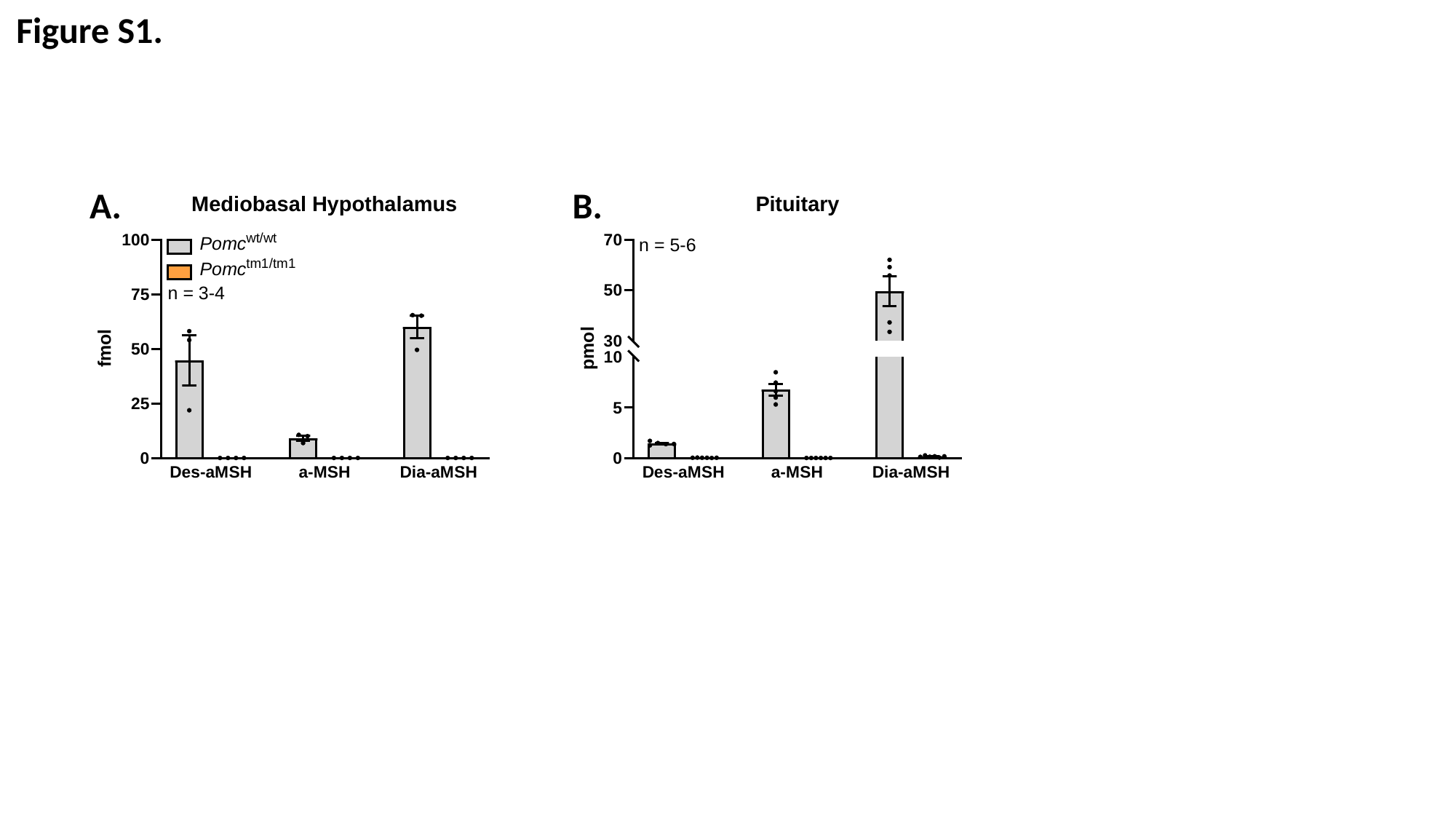

Figure S1.
A.
B.

## Slide 2
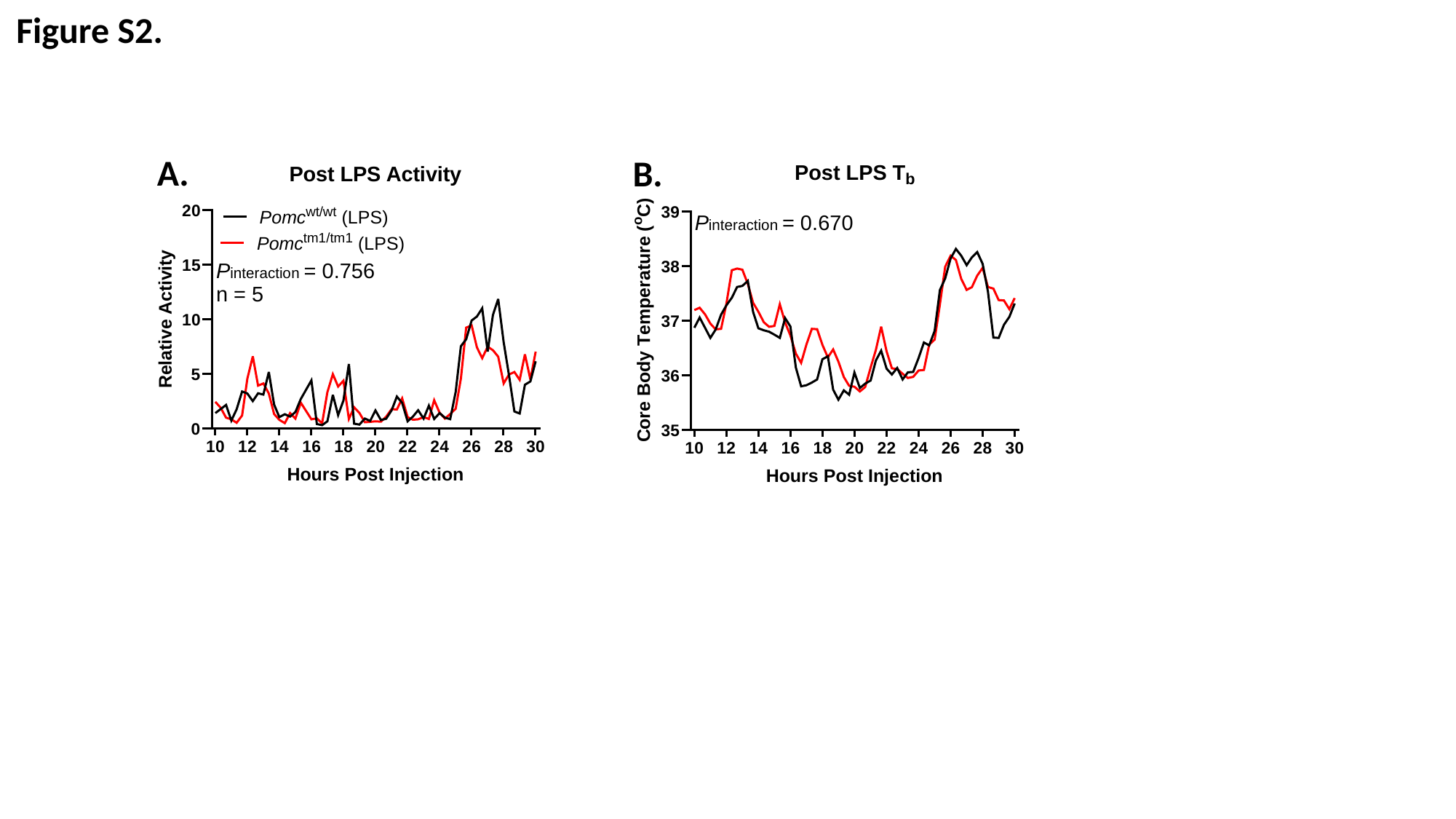

Figure S2.
A.
B.

## Slide 3
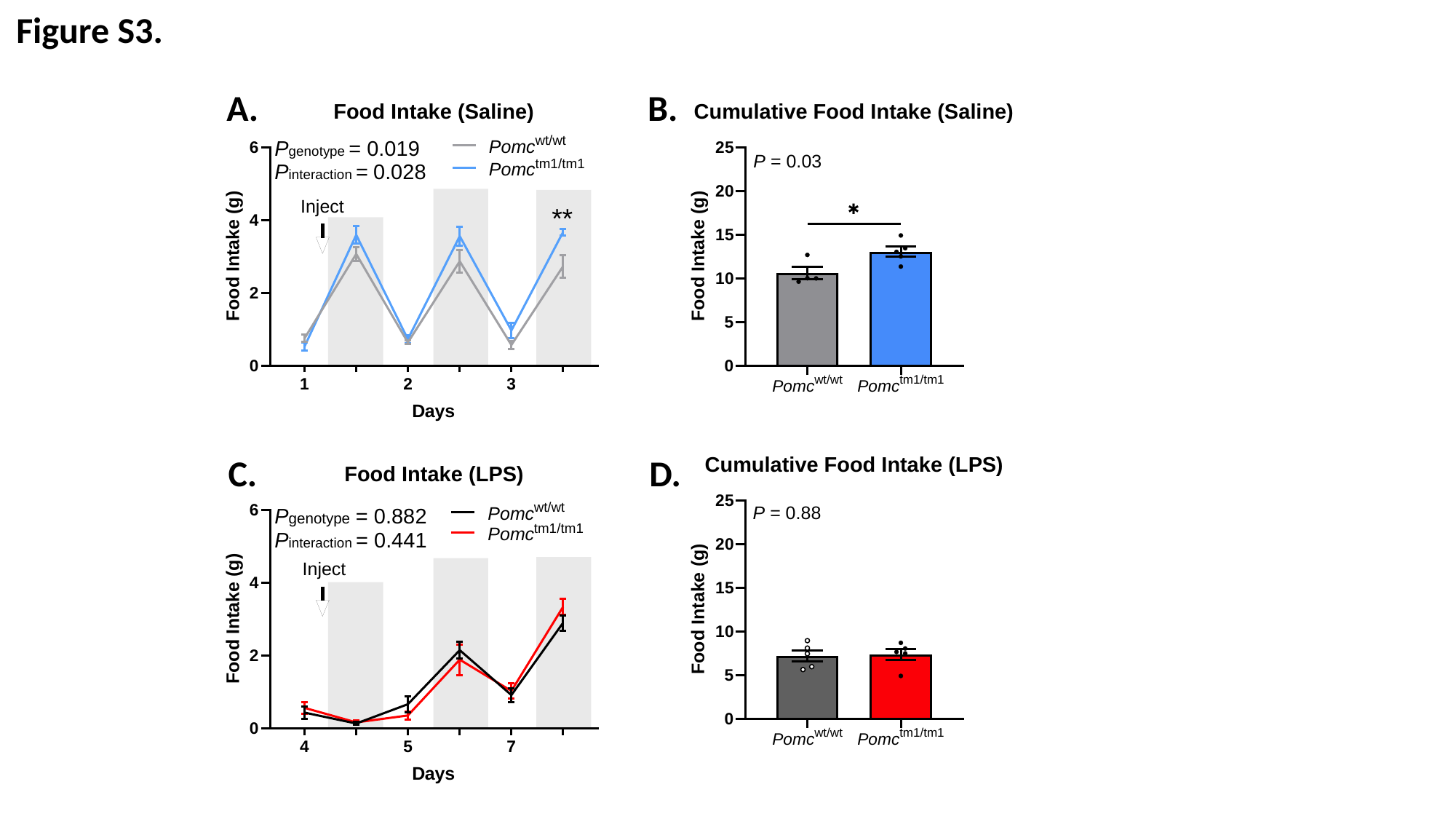

Figure S3.
A.
B.
C.
D.

## Slide 4
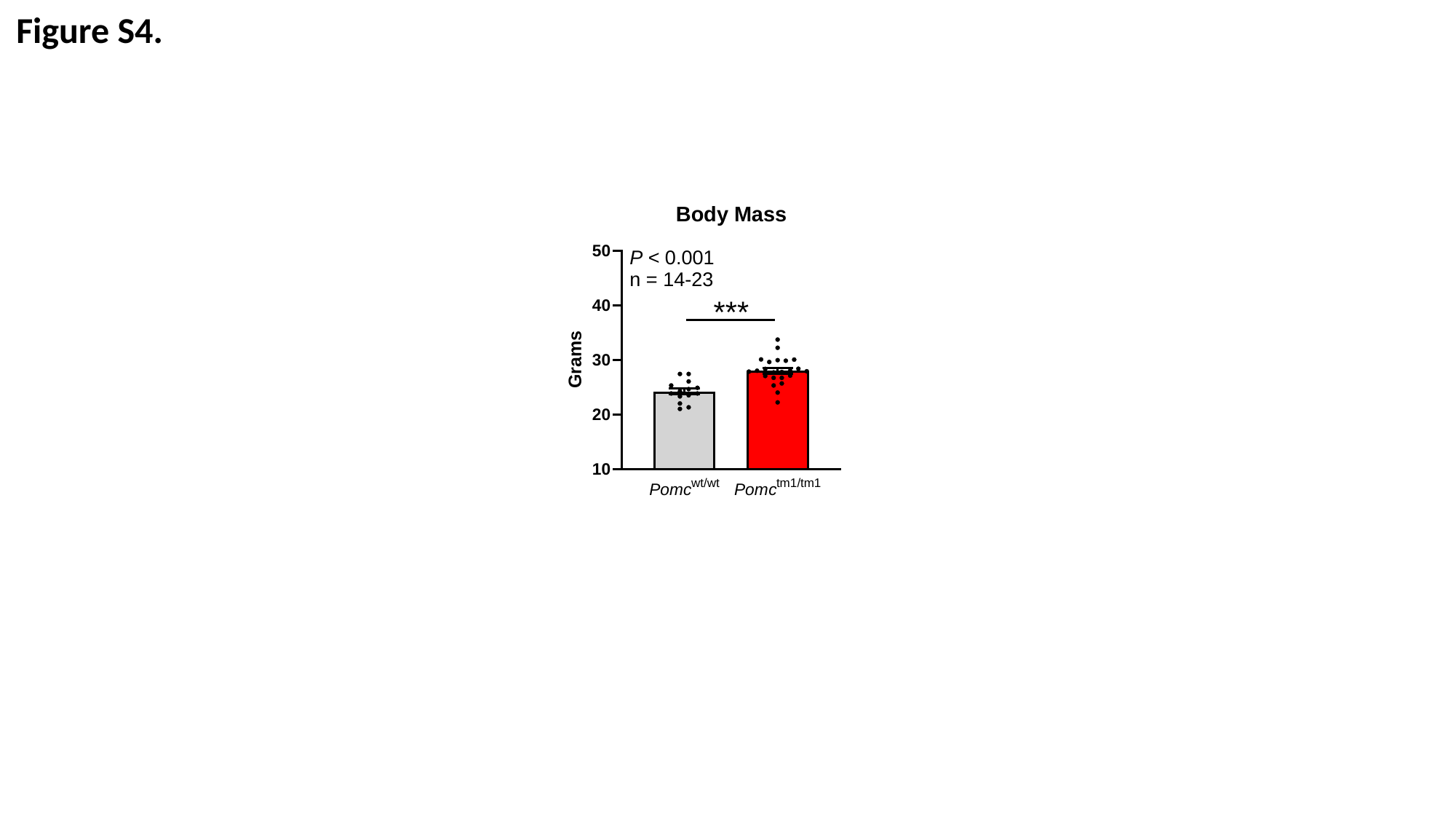

Figure S4.

Supplement: Multimedia component 2 [file mmc2.pptx]
