# Supplementary material for: Alpha-melanocyte-stimulating hormone contributes to an anti-inflammatory response to lipopolysaccharide
Source: Mol Metab. 2024 Jul 9;87:101986. doi: 10.1016/j.molmet.2024.101986 (PMC11362619; doi:10.1016/j.molmet.2024.101986)
Supplement: Multimedia component 3 [file mmc3.docx]

**Figure S1. Deletion of α-MSH in *Pomc^tm1/tm1^* mice measured using selective reaction monitoring mass spectrometry analysis.** (A) Two *Pomc^wt/wt^* or *Pomc^tm1/tm1^* mediobasal hypothalami were combined for each sample and analyzed by mass spectrometry. All three α-MSH forms were undetectable in *Pomc^tm1/tm1^* mediobasal hypothalamic samples. (B) Individual whole pituitary glands were collected and analyzed in triplicate by mass spectrometry. The data are expressed as the mean ± SEM. *p<0.05 compared to littermate controls.

**Figure S2. *Pomc*^tm1/tm1^ mice showed normal late-phase activity and T_b_ after LPS injection.** *Pomc*^wt/wt^ and *Pomc*^tm1/tm1^ littermates showed normal **(A)** relative activity and **(B)** core body temperature from 10–30 h post 100 μg/kg i.p. LPS injection.

**Figure S3. *Pomc*^tm1/tm1^ mice had increased food intake at baseline, but similar food intake after LPS injection. (A–B)** *Pomc*^wt/wt^ and *Pomc*^tm1/tm1^ mice were injected with saline on Day 1 and then **(C–D)** LPS on Day 4. Food intake was recorded for 3 days following each injection and cumulative totals for Saline and LPS were calculated over the 3-day period. The data are expressed as the mean ± SEM and compared to littermate controls.

**Figure S4. *Pomc*^tm1/tm1^ mice have increased body mass compared to *Pomc*^wt/wt^ littermates.** The data are expressed as the mean ± SEM and compared to littermate controls.

**Table S1. Summary of statistics.**

**Table S2. Summary of statistics for cytokine assays per time point.** Outliers were identified and removed from analysis, and Šídák's multiple comparisons test was performed for cytokines with significant interaction effects (shown in Table S1).
